# Barriers and facilitators in implementing population based common cancer screening through community health workers

**DOI:** 10.3332/ecancer.2021.1277

**Published:** 2021-08-19

**Authors:** Ashwini Kedar, Amrita John, Subhadra Goala, Roshni Babu, Ritesh Tapkire, Ravi Kannan, Roopa Hariprasad

**Affiliations:** 1 National Viral Hepatitis Control Program, Ministry of Health and Family Welfare, Nirman Bhawan, Delhi 110001, India; 2 Guntherstrase, 34, Duisburg, North Rhine Westphalia, 47051, Germany; 3 Cachar Cancer Hospital and Research Center, NS Avenue, Meherpur, Silchar 788015, Assam, India; 4 Fogarty International Fellow, Yenepoya University, University Road Deralakatte Mangalore 575018, Karnataka 575022, India; 5 Division of Clinical Oncology, Indian Council of Medical Research-National Institute of Cancer Prevention & Research (ICMR-NICPR), I-7 Sector 39, Noida 201301, India

**Keywords:** community health workers, cancer screening and predetection, barriers, facilitators, qualitative study, common cancer screening

## Abstract

Population based cancer screening was initiated in India in 2016 owing to an increased burden of cancers. A feasibility health system study was done by utilising community health workers (CHWs) to conduct the cancer screening. The current study is a qualitative study to elicit the barriers and facilitators in implementing population based cancer screening through CHWs. The study was conducted at three subcentres of Dholai block of Cachar district, Assam, India and Cachar Cancer Hospital and Research Center, Silchar. The participants of the study were CHWs, master trainer nurses and women from community. Three focus group discussions (FGDs) and one in-depth interview (IDI) were conducted at the provider level and seven IDIs of women from the community. The FGDs and IDIs were audio recorded after taking verbal consent from the participants. The verbatims were prepared following translation and transcription and data analysis using ATLAS ti ver 8. The major barrier faced by the community was a lack of motivation to get screened which stemmed from various factors such as personal beliefs, attitudes and fear. The major facilitators were accessibility of tests, family support and CHWs as screening service providers. The major barriers for CHWs were difficulty in motivating the community, lack of support from supervisors and lack of motivation to work. The major facilitators were convenience of screening during home visits, empowerment, skill enhancement and teamwork. Population based cancer screening was a new concept for the community under study. Cancer screening by CHWs was well accepted by the community. Awareness generation among the community was a major factor in improving screening coverage. The study highlights that training and motivation of CHWs can improve the uptake of cancer screening services. CHWs felt empowered with the new skills imparted and were able to carry out screening.

## Introduction

The burden of cancer has been increasing in India since the past few decades. Westernisation and urbanisation have led to tremendous lifestyle changes and with changing population demographics of India, the incidence of cancer is increasing. Among all the cancers in India, oral cancer, breast cancer and cervical cancer are the most common cancers which are preventable and can be easily detected in its early stages [1]. Hence, in 2016, Government of India (GOI) launched the operational guidelines for population-based cancer screening of the three common cancers under the National Program for Prevention and Control of Cancer, Diabetes, Cardiovascular Diseases and Stroke [2,3]. As per the guidelines, 30–65 years old population will be eligible for screening and screening will be done by the Auxiliary Nurse Midwife (ANM) or nurse posted at the subcentre (SC).

An implementation research was undertaken to check the feasibility of this population-based cancer screening guidelines in the community. As the community under study was being exposed to cancer screening tests for the first time, the study was conducted after each of the communities had received knowledge and awareness by the community health workers (CHWs) for at least 3 months. Two changes were made during implementation: 1). population-based cancer screening was done by trained Accredited Social Health Activist (ASHA) who is equivalent to a CHW and 2). Screening was conducted during home visits. The current study was undertaken to assess qualitatively the various barriers and facilitators in the implementation of the programme at the level of community and health care providers (HCPs).

## Methods

### Study setting

The common cancer screening was implemented in the population catered by the 45 SCs under Dholai Block, Cachar district in Assam, India by the CHWs from January 2018. A SC is a public health facility under Indian Public Health System which is nearest to the community. The SC is manned by ANMs. Each SC caters to a population of 5,000. ASHAs, i.e., CHW links the community with public health facilities. Each ASHA caters to a population of 1,000 and helps the respective ANMs of the SC for healthcare service delivery. ASHAs provide services for various national health programmes and scheme under the broad umbrella of National Health Mission, India. She is incentivised for every service she provides. Incentives were provided to the ASHAs for the complete screening and data entry of each member of eligible population covered under the study.

The current study was conducted at three SCs: Palanghat, Bagbahar, Basknal Puttikal and at Cachar Cancer Hospital and Research Center (CCHRC), Silchar, Assam. Only those SCs were selected for the study where at least 1 month had passed following the training of CHWs. The study was conducted between October 2018 and February2019.

### Study design

A qualitative design was selected for this study to elicit in detail the various barriers and facilitators in implementation of the common cancer screening in the community at the level of CHWs, master trainer nurses (trained the CHWs) and community women.

### Sampling method and data collection

The SCs were selected purposively. Most CHWs of the SCs had received training by June 2018. The Focus Group Discussions (FGDs) were planned at two SCs. The selection was based on the population coverage for screening. In the initial 6 months, the CHWs had to screen 50% of the eligible population. One SC had a good cancer screening coverage and had achieved the target, whereas the other had not achieved and had poor coverage. An in-depth interview (IDI) was planned with a CHW working at Bagbahar as she was one of the CHWs who had accomplished good coverage of cancer screening services in her area and was being trained to be master trainer for training other CHWs.

Another FGD was conducted with the master trainers of CCHRC as they were training the CHWs and were working closely with them in the community.

IDIs were conducted with seven women from the community at the specific SCs. They were purposively selected as few had refused screening by CHWs and few had accepted the services provided by her.

The interviews were conducted by researchers (BR, HR) from the National Institute of Cancer Prevention and Research (NICPR) Noida and research officer from CCHRC (GS) who was well versed in the local language and accompanied them to double translate the interview. The interviewers had prior experience in conducting qualitative research. Considering the nature of the questions and to elicit adequate information, female interviewers were selected to conduct the interviews. FGDs were held with CHWs and master trainers by two researchers from NICPR (KA, HR).

The details of the data collection are presented in Table 1.

The FGDs and IDIs were audio recorded after taking verbal consent from the participants. The interview was conducted in local language (Bengali) which were translated into a language the researchers were familiar with and field notes were taken by the interviewers. The data collection for the current study was done between November 2018 and January 2019.

### Data collection tool and procedures

FGDs and IDIs were done using the discussion guides covering specific topics.

To ensure validity of data, most questions in the interview and FGD were open ended questions and appropriate probes were used where required.

### Data analysis

The recordings of FGDs and IDIs were translated and transcribed immediately after the data collection. Three authors went through the transcribed data in detail to ensure completeness, accuracy and to elicit the emerging codes. The emerging codes were formulated separately by each of the three authors following which the codes were finalised in consensus. Codes were made and evaluated in view of the study objectives, interview, FGD guides and the data.

Data analysis was done using the ATLAS.ti Ver 8. Each FGD and interview was entered into the software as an individual document and thematic content analysis was used to analyse the data. Each document was read line by line and appropriate codes were assigned to the relevant data segment. In-vivo coding was also done when the authors felt that a new code needs to be assigned apart from the code book.

### Ethical considerations

The study was approved by ethical committee of NICPR, Indian Council of Medical Research (ICMR). Prior to each FGD and interview, a written informed consent and an audio consent were taken from each participant.

## Result

### Demographic details of participants

The participants from the community were seven women with age ranging from 30 to 45 years. Majority of them were homemakers with few involved in local farming practices. All had attended schooling till primary grade.

The nine CHWs in the two focus groups had an age range from 35 to 54 years with majority having educational qualification till 10th grade. The years of work experience as CHWs ranged from 4 to 15 years.

The seven master trainers in the FGD were in the age range of 26–34 years and were graduates with BSc nursing as qualification. The work experience as a nurse varied from 3 to 12 years.

*Barriers and facilitators*

The barriers and facilitators found in the datasets of community and HCPs are being presented in an integrated form and have been detailed separately in Tables 2 and 3.

#### Barriers

Most participants were in favour of the community based cancer screening but few barriers were noted in the implementation process and there was an initial resistance in the community to accept screening services. One of the main reasons to refuse screening was absence of any visible signs/symptoms of any health problem.

*…I don’t have any cervical problem at present. I have my gynaecologist at Lifeline hospital to whom I regularly visit. If there is any problem I go there…. as my sons and daughters are adults now, hence I do not want to get screened …I have no problem, why should I get test done? (P 6)*

*….they don’t listen to us….even if we inform them, they say they don’t want anything from us…they tell us to go away….this has happened many times with me….they tell me that they will go to the hospital themselves if they have any problem and they don’t want anything from me..FGD3*

A belief that cancer is contagious and deadly has also created a fear among the community and CHWs which was a demotivating factor.

*..few people feel scared of us as we are from the cancer program. They don’t want to get tested as they feel that they don’t have any cancer…FGD 2*

*..they (CHWs) learn the skills but they don’t want to screen as they think that if they screen they will also get cancer…FGD3*

These reasons mainly stemmed from a lack of knowledge and awareness among the community regarding cancer screening. Hence, CHWs and master trainers found it difficult to motivate the community for screening and community required a number of counselling and awareness sessions prior to initiation of screening.

*Initially when I started it was very difficult to make people understand to get screening done as these people stay in tea gardens and are illiterate and it is very difficult to make them understand A1*

*The part of convincing the population for screening was difficult …when we go to rural area it is very difficult to convince the lady…even after we make them understand they don’t agree for screening...FGD3*

*We have to do counselling…when we visit homes first time they don’t know why we have come hence we counsel, then they get screened after 2–3 months….if they are aware then they agree quickly for the tests so when we go for the first time many don’t get screened..FGD3*

Few other barriers among community was mainly related to cervical cancer screening which was due to feeling shy to get tested or discomfort experienced by the women who were screened.

*…A little pain was there in cervical test...(P 2)*

*…..I felt a burning sensation when medicine was applied on cervix… (P 4)*

*…. Yes I felt a little hesitation and shyness…P3*

At times some coincidental events were linked falsely as a consequence of the cervical screening test which led to discordance among community and CHWs.

*….once we did a camp at a school; a woman had come with complaint of white discharge so we did the VIA and after that her white discharge increased following which she held all of us responsible... (FGD 3)*

Some communities did not trust the CHWs and harbour misconceptions about the screening tests.

*Some people in the field have false notions and think that we have come to kill their children….some women think that when we insert speculum we are putting virus into them…..some people say that we are doing some test so that they don’t have more children...some said that sterilization procedure is being done.. FGD 3*

Barriers in the implementation process for CHWs were nonsupportive supervisors who would engage them in other public health related work.

*They (ANM) don’t let us stay in peace even for a single day. Didi (ANM) orders us to take reports, papers and to get it back; to take iron to school for distribution. Since 2 months this is going on. ANM said that we cannot screen the patients at subcenter (FGD1)*

ASHAs were given incentives for the cancer screening services and the data collection for each eligible population. However, few ASHAs perceived the incentives to be meagre which was a demotivating factor for carrying out screening.*.I think the incentive we (CHW) are getting is a little less…we should get Rs.20 for screening one individual FGD 2*

*Many CHWs have a problem with incentives…..they think that the incentive they get is a little less ..initially during training they (CHW) would tell us that they will do the screening….now when they have started doing it they feel it is less …they say as to what they will get by doing this work… (FGD 3)*

Few CHWs felt overburdened with the work of screening and found it difficult to manage their time with other work.

*We are not getting time to do screening…we have other work like vaccination, distribution of iron tablets, ANC visits of pregnant women...we have to go to subcenter daily except Sunday FGD 2*

The master trainers found a few barriers in training the CHWs such as language barrier, lack of interest of CHW in learning new skill and difficulty in learning cervical screening test and entry on tablets.

*The CHWs don’t know the medical part of the screening which is new for her. They mostly deal with maternity cases. Screening is completely new for them. So, they need to learn theory and practical such as BP, Sugar. We are able to teach them…. There was language problem if they are tribal as they don’t know Bengali. (FGD 3)*

*Teaching them (CHWs) the cervical screening was difficult…when we tell them speculum insertion, they are not able to understand or grasp soon (FGD 3)*

*Training on tablets – there is a lot of problem there…some CHWs find it problematic on seeing it…few CHWs have never used a smartphone hence this is a problem for them…hence we ask CHW if they have a young member in family familiar with smart phone either daughter, husband, brother and tell them to come during training and we train them on tablets… t the entry on tab, i.e. filling up the name, etc. is confusing for her (CHW). …she is able to do …but takes time (FGD3)*

#### Facilitators

Presence of knowledge and awareness of cancer and presence of symptoms increased the acceptability of cancer services.* I came by myself to get the test done as I had backpain and pain during intercourse….P2*

*….. I called CHW and told that I am having itching in lower part and I want to undergo these tests……my neighbour also had a problem in mouth of uterus thus she also agreed to get these tests done....P3*

Presence of family support was a motivator for accepting the screening tests.

*…Yes my family supported and no one objected…..My sister in law).. ….she also got her screening done at subcenter after me…Yes, CHW as well as her husband (my brother) told her (sister in law) to get herself screened. P2*

People who experienced the screening tests became ambassadors for the same and promoted ‘word of mouth’ information.

*…I heard about these tests from people staying in a distant area. The CHW working in our area had not started the screening in my area but I wanted to get the tests done….. I also told my neighbour….. I told her to get these tests done and today she has come….if someone tells me that they have breast or cervix problem.., I tell them to get these tests done. P3*

The acceptance and respect for CHW and the counselling provided by them led to better acceptance of services.

*…All the women and CHW were there in the house where test was being done. But in the room where examination was being done only I and CHW was there…P1*

*They(community) trust us (CHWs)……we take care of the women during pregnancy and delivery, nursing … so she trusts us. community has accepted us well…FGD2*

The accessibility of services as per the comfort of the community was also a major facilitator.

*…Didi (CHW) checked my cervix at home on my bed and I was comfortable… no I did not feel any pain.. P5*

*… CHW told me I have to go to cancer hospital. and I underwent a test and was seen through a machine…P3*

Major facilitators for CHWs to carry out screening were the option of screening at home during home visits.

..*when we (CHW) do home screening we can do other work also allotted to us. Madam even I like to go for home visit. When I go for injection then I do the screening also. (FGD 1)*

Teamwork among CHWs by helping each other in screening led to better coverage.

*…mostly out of 5 CHWs , 3 CHWs learn quickly…2 CHWs find it difficult so we advise them that they go in community together…those who are expert in making acetic acid that person should make the same , the one who is expert in writing may write and one who is good in speculum insertion does the same…so when we are there with them we teach them everything…..but we want them to work after we leave….so those who work together we advise them to choose one task they are good at and screen in a team…and cover one area at a time (FGD 3)*

A feeling of empowerment, skill enhancement and recognition for work was major motivating factor for CHWs.

*I found it good. I learnt a lot. I did not know how to wear gloves. I did not know anything about these tests…….When I started in the beginning I had a lot of problems learning…I had given up. One day master trainer held my hand and told me why I cannot learn and told me to try. (A1)*

*In a meeting we awarded and appreciated those CHWs who were doing screening very well…. …we hope that other CHWs also do well to receive an award next time. (FGD 3)*

Supportive supervisors were important facilitators to carry out the cancer screening along with other public health related work.

*yes she(ANM) supports us..she stays here at the subcenter…we take the vaccines with her from here for immunization (FGD 2)*

*Those who have supportive ANM they were able to do good (FGD 1)*

## Discussion

The population based cancer screening programme in India is being implemented in a phased manner. As per the Indian guidelines, cancer screening will be done by ANM at SCs unlike in this study where CHWs are screening the community during home visits. CHWs are a large workforce in the Indian public health system, bridging the gap between the community and health care facilities. Screening is a relatively new concept for Indian adults.

Literature review provides data on few studies where community based cancer screening was done by CHWs mainly involving either breast, cervical or oral cancers. This is one of the first implementation researches in India where CHWs conduct community based screening of all the three cancers. The implementation research attempts to introduce screening utilising the already existing public health system workforce. This qualitative study has made an attempt to bring forth the various barriers and facilitators in the initial phase of screening and provides rich perspective of the community and HCPs.

The community and CHWs where screening started was naïve to such a process. Awareness generation was crucial for the community and CHWs. Following this, the community based screening became more acceptable. A study from Nepal showed that misunderstanding and lack of knowledge were found to be main barriers to cervical cancer screening [4].

Motivating the community for screening was one of the difficult tasks faced by CHWs. One of the common reasons was absence of any symptoms. The community people were yet to accept screening as a preventive measure and considered going to hospital in case of health problems. Creating awareness helped the community accept the process. Similar findings were seen in other studies [4, 5].

An inherent fear of cancer was present among community and CHWs, which hindered the process of screening. A similar perception of fear where cancer was associated with dread and death was observed in other studies globally and in India [6]. These negative fatalistic perceptions have been observed to be barriers to uptake of cancer screening and for people to follow preventive behaviours [7, 8].

Unsupportive supervisors, competing public health tasks and unfounded community rumours created hurdles to CHW participating in the study and hence requiring education, time management and reassurance to be provided by the master trainers.

Most community women accepted the service provided by CHWs and found it more acceptable and accessible. A systematic review on CHWs in cervical cancer screening showed that involvement of CHWs was largely acceptable and feasible [9]. Women who had prior knowledge of cancer through awareness programmes tended to accept screening services.

Family support in the homes of the women in the community, possibly fostered in part by word of mouth support from those who had already been screened encouraged women to agree to being screened. This coupled with the fact that screening was being performed by a familiar member of the community (CHW) and at home at the woman’s convenience helped the programme to succeed.

Learning about cancer, its prevention and screening, the ability to implement this efficiently in the community, acceptance by the community of the CHWs in this role, the satisfaction of having been able to save lives by screening and the confidence in the back up provided by the master trainers resulted in the CHWs feeling empowered and respected. This possibly improved their self-esteem and was a major motivator for the CHWs to participate.

Target linked financial incentives were another major motivator. Though some CHWs felt the quantum of incentive to be low, most were happy with it.

Encouraging CHWs to work in groups augmenting each other’s skills, providing recognition to CHWs who performed well and offering support to CHWs by attending to their screening related issues appear to have facilitated the process. CHWs who had supportive ANMs in the SC also appeared to be more committed.

### Limitations

Qualitative study may not be generalisable to a larger population. However, we have kept robust methodological design and ensured a minimum number of participants for each stakeholder group, which were all analysed and a theoretical saturation was obtained [10, 11].

This study did not include male population.

### Strengths

This qualitative study tries to encompass views of all participants, community as well as the HCPs involved in the population based cancer screening. Eight interviews and three FGDs were sufficient to elicit the barriers and facilitators of the programme.

## Conclusion

The current study highlights the barriers and facilitators of implementing population based cancer screening during home visits by CHWs. The study highlights that dissemination of information about cancer screening for the community along with training and motivation of the CHWs can improve the uptake of services. Adequate support in the form of reassurance and follow through, incentives and provision of time and place for performing screening will aid the CHWs to perform their duties more efficiently. Our study showed that population based cancer screening was accepted better by the community as it was implemented with the help of CHWs who were doing screening at the community level.

## List of abbreviations

GOI, Government of India; ANM, Auxiliary nurse midwife; SC, Subcentre; CHW, Community health worker; ASHA, Accredited Social Health Activist; HCP, Health care provider; CCHRC, Cachar Cancer Hospital and Research Center; FGD, Focus group discussions; NICPR, National Institute of Cancer Prevention and Research; ICMR, Indian Council of Medical Research, ANC, Ante natal care.

## Competing interests

None.

## Ethics approval statement

Study has been approved by the Institutional Ethical Committee of CCHRC.

## Contributorship statement

AK: Data collection, data transcription, coding and analysis, manuscript drafting editing. AJ: Data transcription, coding and analysis, manuscript drafting and editing. SG: Data collection, data transcription, manuscript drafting and editing. RB: Data collection, data coding, analysis and manuscript drafting and editing. RT: Data analysis, manuscript drafting and editing. RK: Implementing the study, data analysis, manuscript drafting and editing. RH: Conception and design of the study, data collection, manuscript editing, procured the funding and approval of final version.

## Funding

Funding received from the ICMR.

## Figures and Tables

**Table 1.** Details of the interviews and FGDs.

	Number of participants	Cancer screening localisation	Duration of interview
FGD^a^ 1	5 CHW	Palanghat Subcentre	51 Minutes 53 seconds
FGD 2	4 CHW^b^	Basknal Puttikal Subcentre	40 Minutes 25 seconds
FGD 3	8 Master trainers	CCHRC	1 hour 4 minutes
IDI^c^-A	1 CHW	Bagbahar Subcentre	21 Minutes 58 seconds
IDI-1	1 woman – screened by CHW	Bagbahar Subcentre	12 minutes 38 seconds
IDI-2	1 woman – screened by CHW	Palanghat Subcentre	10 minutes 55 seconds
IDI-3	1 woman – screened by CHW	Palanghat Subcentre	11 minutes 43 seconds
IDI-4	1 woman – screened by CHW	Palanghat Subcentre	10 minutes 21 seconds
IDI-5	1 woman – screened by CHW	Bagbahar Subcentre	11 minutes
IDI-6	1 woman – refused cervical screening by CHW	Basknal Puttikal Subcentre	9 minutes 42 seconds
IDI-7	1 woman – refused cervical screening by CHW	Basknal Puttikal	11 minutes 32 seconds

a FGD, Focus group discussion

b CHW, Community health worker

n IDI, In-depth interview

**Table 2.** Barriers elicited following analysis of transcribed verbatim.

Barriers	Occurrence of themes
**Women from community**	
Problem during screening process Pain during cervical test Burning sensation during cervical test	1 1
Lack of motivation for getting screened Absence of any visible signs/symptoms of health problem Personal attitudes – shy to get tested the private parts Fear of word ‘cancer’ Personal beliefs – religious belief Preference of testing by doctor at hospital	3 2 2 2 3
**CHW (FGD)**	
Difficulty in motivating community for screening	10
Women are shy	4
Refusal for screening by community Fear of cancer Fear of hospitals	2 3
Lack of free transportation for referred patients	2
Presence of family members at patients home	4
Lack of support from supervisors and higher officials	6
Attitude to postpone work	7
Other public health work	8
Insufficient incentives	9
**Master trainers (FGD)**	
Difficulty in motivating community	12
Lack of knowledge and awareness in community	11
Issues in training and motivating CHWs to work	14
Refusal by CHWs to work Lack of interest Old CHWs with health issues Fear of cancer Inadequate incentive Overburdened with other work	4 3 2 5 5
CHWs feel overburdened	5
Refusal of services by community Rich people do not want services from CHW Coincidental events with screening Wrong beliefs about cervical screening tests Fear of cancer Fear of gloves and equipment	3 4 5 3 2
Difficult geographical terrain	3
Community expectations	2

**Table 3.** Facilitators elicited following analysis of transcribed verbatim.

Facilitators	Occurrence of themes
**Women from community**	
Individual facilitators Knowledge and awareness on symptoms and sign of cancers Presence of symptoms Family support	6 3 5
Screening by CHW Appropriate counselling and information given by CHW Provision of privacy Post-test counselling by CHW Acceptance and respect for CHW (helping also)	5 4 3 6
Accessibility of tests Comfort of getting tested at home or nearby area Well-established referral system	8 3
‘Word of mouth’ screening	7
**CHWs (FGD)**	
Convenience of screening during home visit	8
Financial incentives	4
Community facilitators	
Trust of people Community acceptance Educated community	4 6 2
Personal factors Dedication to community work Skill enhancement Empowerment Personal acceptance and experiencing screening	2 3 3 1
Supportive supervisors	2
Supportive master trainers	3
**Master trainers (FGD)**	
Teamwork among CHWs	6
Feeling of empowerment among CHWs	3
Giving recognition to CHWs for the work	3
Help by ANM	2
Screening among CHWs	4
